# EuroFlow Standardized Approach to Diagnostic Immunopheneotyping of Severe PID in Newborns and Young Children

**DOI:** 10.3389/fimmu.2020.00371

**Published:** 2020-03-19

**Authors:** Tomas Kalina, Marina Bakardjieva, Maartje Blom, Martin Perez-Andres, Barbara Barendregt, Veronika Kanderová, Carolien Bonroy, Jan Philippé, Elena Blanco, Ingrid Pico-Knijnenburg, Jitse H. M. P. Paping, Beata Wolska-Kuśnierz, Malgorzata Pac, Jakub Tkazcyk, Filomeen Haerynck, Himmet Haluk Akar, Renata Formánková, Tomáš Freiberger, Michael Svatoň, Anna Šedivá, Sonia Arriba-Méndez, Alberto Orfao, Jacques J. M. van Dongen, Mirjam van der Burg

**Affiliations:** ^1^Department of Paediatric Haematology and Oncology, Second Faculty of Medicine, Charles University and University Hospital Motol, Prague, Czechia; ^2^Laboratory for Immunology, Department of Pediatrics, Leiden University Medical Center (LUMC), Leiden, Netherlands; ^3^Department of Medicine-Serv. Cytometry, Cancer Research Center (IBMCC-CSIC/USAL), University of Salamanca, Salamanca, Spain; ^4^Department of Immunology, Erasmus MC, University Medical Center Rotterdam, Rotterdam, Netherlands; ^5^Department of Diagnostic Sciences, Ghent University, Ghent, Belgium; ^6^Department of Laboratory Medicine, Ghent University Hospital, Ghent, Belgium; ^7^Department of Immunology, Children's Memorial Health Institute, Warsaw, Poland; ^8^Department of Pediatrics, Second Faculty of Medicine, Charles University and University Hospital Motol, Prague, Czechia; ^9^PID Research Lab, Department of Pediatric Pulmonology and Immunology, Ghent University Hospital, Ghent, Belgium; ^10^Department of Pediatric Immunology and Allergy, Kanuni Sultan Süleyman Training and Research Hospital, Istanbul Health Sciences University, Istanbul, Turkey; ^11^Centre for Cardiovascular Surgery and Transplantation, Brno, Czechia; ^12^Medical Faculty, Masaryk University, Brno, Czechia; ^13^Department of Immunology, University Hospital Motol, Prague, Czechia; ^14^Servicio de Pediatría, Hospital Universitario de Salamanca, Salamanca, Spain; ^15^Department of Immunohematology and Blood Transfusion (IHB), Leiden University Medical Center (LUMC), Leiden, Netherlands

**Keywords:** flow cytometric immunophenotyping, primary immunodeficiencies (PID), EuroFlow, standardization, severe combined immune deficiency (SCID), diagnosis

## Abstract

The EuroFlow PID consortium developed a set of flow cytometry tests for evaluation of patients with suspicion of primary immunodeficiency (PID). In this technical report we evaluate the performance of the SCID-RTE tube that explores the presence of recent thymic emigrants (RTE) together with T-cell activation status and maturation stages and discuss its applicability in the context of the broader EuroFlow PID flow cytometry testing algorithm for diagnostic orientation of PID of the lymphoid system. We have analyzed peripheral blood cells of 26 patients diagnosed between birth and 2 years of age with a genetically defined primary immunodeficiency disorder: 15 severe combined immunodeficiency (SCID) patients had disease-causing mutations in *RAG1 or RAG2* (*n* = 4, two of them presented with Omenn syndrome), *IL2RG* (*n* = 4, one of them with confirmed maternal engraftment), *NHEJ1* (*n* = 1), *CD3E* (*n* = 1), *ADA* (*n* = 1), *JAK3* (*n* = 3, two of them with maternal engraftment) and *DCLRE1C* (*n* = 1) and 11 other PID patients had diverse molecular defects [*ZAP70* (*n* = 1), *WAS* (*n* = 2), *PNP* (*n* = 1), *FOXP3* (*n* = 1), del22q11.2 (DiGeorge *n* = 4), *CDC42* (*n* = 1) and *FAS* (*n* = 1)]. In addition, 44 healthy controls in the same age group were analyzed using the SCID-RTE tube in four EuroFlow laboratories using a standardized 8-color approach. RTE were defined as CD62L+CD45RO-HLA-DR-CD31+ and the activation status was assessed by the expression of HLA-DR+. Naïve CD8+ T-lymphocytes and naïve CD4+ T-lymphocytes were defined as CD62L+CD45RO-HLA-DR-. With the SCID-RTE tube, we identified patients with PID by low levels or absence of RTE in comparison to controls as well as low levels of naïve CD4+ and naïve CD8+ lymphocytes. These parameters yielded 100% sensitivity for SCID. All SCID patients had absence of RTE, including the patients with confirmed maternal engraftment or oligoclonally expanded T-cells characteristic for Omenn syndrome. Another dominant finding was the increased numbers of activated CD4+HLA-DR+ and CD8+HLA-DR+ lymphocytes. Therefore, the EuroFlow SCID-RTE tube together with the previously published PIDOT tube form a sensitive and complete cytometric diagnostic test suitable for patients suspected of severe PID (SCID or CID) as well as for children identified via newborn screening programs for SCID with low or absent T-cell receptor excision circles (TRECs).

## Introduction

Severe combined immunodeficiency (SCID) and combined immunodeficiency (CID) are two of the most severe forms of inherited disorders of the immune system (1, 2) with an incidence of 1:35,000–50,000 newborns. Patients are usually born asymptomatic, but they develop severe (opportunistic) infections, failure to thrive within the first months of life and generally die before the age of 1 year, unless they receive adequate and curative treatment. This includes hematopoietic stem cell transplantation (HSCT). For some genetic forms of SCID gene therapy is available (3, 4). HSCT is indicated immediately after birth, since patients transplanted before the age of 3.5 months or patients without infections have a superior prognosis as compared to those transplanted later or when infectious complications have accumulated (5, 6). In contrast, patients with CID usually do not have complete absence of T-lymphocytes as typically seen in SCID, but they frequently show profound impairment of T-cell immunity leading to severe infections, autoimmunity, and malignancies. Thus, the indication of HSCT for CID is less clear as it is less evident whether the T-cell deficiency is sufficiently severe to justify the risks of HSCT (7).

T-cells are generated in the thymus and released to peripheral blood as antigen inexperienced, naïve T-cells. These cells called “recent thymic emigrants” (RTE) are the recently formed naïve T-cells that are produced in the thymus and their numbers correlate with thymic output (8). To date, disease-causing mutations have been reported in 17 genes leading to SCID, and another 43 genes are reported as being mutated in CID (9, 10). The majority of SCID and CID patients presenting in the first 2 years of life have a defect in T-cell development in the thymus. A complete defect (null mutation) results in absence of T-cells, but hypomorphic (“leaky”) mutations can give rise to an incomplete defect leading to presence of variable numbers of T-cells with poor immune function and inadequate control of autoreactivity. This leads to immunodeficiency and dysregulation such as seen in Omenn syndrome (11). Likewise, variable degree of T-cell immunodeficiency is found in patients diagnosed with 22q11.2 deletion syndrome (DiGeorge syndrome) (12).

An assay for early detection of SCID via newborn screening (NBS) has become available to identify T-cell lymphopenia directly after birth. This assay is based on measurement of T-cell receptor excision circles (TRECs) via quantitative PCR on dried blood spots (13). TRECs are formed as circular excision products during T-cell receptor gene rearrangement in developing T-cells in the thymus and are a molecular marker for recently formed T-lymphocytes. Absence or strongly reduced levels of TRECs are indicative for T-cell lymphopenia and can identify children who may have SCID. TRECs will not be detected in case of the presence of maternally engrafted T-cells. In addition, TRECs will also be low/absent in patients with Omenn Syndrome because of oligoclonal expansion of the autologous T-cells, which makes the TREC assay also useful in these subtypes of SCID. Follow-up diagnostic testing in case of low or absent TREC contents is needed to confirm the diagnosis by flow cytometric immunophenotyping and subsequently by targeted genetic testing for SCID-CID gene aberrations or broader genetic testing (e.g., WES or WGS) in combination with a SCID or PID filter. It should be noted that low or absent TRECs can also be identified in children with T-cell impairment syndromes [such as 22q11.2 deletion syndrome (14), Down's syndrome or Ataxia Telangiectasia], and children with T-cell impairment secondary to other neonatal conditions or patients with idiopathic lymphocytopenia (15, 16). Furthermore, low/absent TREC levels can also be found in preterm children or in children from mothers on immunosuppressive therapy (17).

Flow cytometric immunophenotyping of lymphocytes proved useful for the early diagnosis of SCID in patients with clinical symptoms or newborns with low/absent TRECs, showing complete lack of one or more lymphocyte lineages (T-cell, B-cell and NK cell) (15, 18). However, interpretation of this basic flow cytometric screening is not sufficient when T-cells are present, either due to a hypomorphic defect or due to the presence of maternal T-cell engraftment. Maternal T-cell engraftment is a relatively frequent finding in SCID (40% in a cohort of 121 patients from Ulm or 47% in the California cohort) (19, 20). In those cases, a flow cytometric test which allows more detailed phenotyping of the T-cells, including analysis of newly generated T-cells, is warranted. At present there is no consensus on the exact composition of a flow cytometric test, although critical parameters (Naïve T-cells, RTE, activated T-cells) are listed by the European Society for Immunodeficiencies (ESID), the American PID treatment consortium (PID-TC) and by other groups (7, 21).

Flow cytometry allows to discriminate naïve T-cell subsets from antigen experienced memory subsets by presence of typical markers (CD45RA isoform, costimulatory molecules CD27, homing receptor CCR7 and CD62L) and absence of memory markers (CD95 and CD45RO isoform) (22, 23). This is useful for diagnostic evaluation of patients with profound T-cell (function) deficiency, where T-cells are detectable (at normal or even increased levels) as a result of peripheral expansion of memory T-cell clones from either autologous or maternal origin. Immunophenotyping could show a skewed redistribution from naïve to memory and activated phenotypes within T-lymphocytes, which alerts for a possible lymphocyte development defect. Furthermore, newly generated T-cells released from the thymus to the periphery (RTE) can also be identified using flow cytometry. RTE are CD4+ T-cells with the highest TREC levels (24) and a phenotype characterized by expression of CD45RA and CD31 (25, 26). Finally, activated T-cells acquire a memory phenotype and temporary signs of activation, which can be detected via analysis of CD69, CD25, and HLA-DR, among other markers (27).

In this study, the EuroFlow PID group consortium has designed, developed and validated a standardized approach for flow cytometric evaluation of naïve, RTE and activated CD4+ and CD8+ T-cells that would offer a high sensitivity test toward disclosing (S)CID in line with the ESID diagnostic criteria in the settings of a multi-center collaboration study. The here developed 8 color “SCID-RTE tube” complements the recently published PIDOT tube (18, 28) for orientation and screening of primary immunodeficiencies (PID) of the lymphoid system. The combination of the two 8-color tubes (or a single 12-color variant of both tubes) could readily be applied in routine diagnostic screening for patients clinically suspected for having (S)CID, as well as in follow-up diagnostics in NBS programs.

## Materials and Methods

### Patient and Control Samples

Our patient cohort consisted of 26 patients with a genetically defined PID diagnosed between birth and the age of 2 years at participating centers (Table 1). Genetic analysis was performed locally according to the routine procedures of the collaborating laboratories using Sanger sequencing or next generation sequencing (NGS). In addition, 44 healthy controls without any known hematological or immunological disorder in the same age range were also enrolled. The samples have been collected from 2013 to 2018. All 26 patient samples were collected according to the local medical ethics regulations of the participating centers, after informed consent was provided by the subjects, their legal representatives, or both, according to the Declaration of Helsinki. The study was approved by the local ethics committees of the participating centers: University of Salamanca, Salamanca, Spain (USAL-CSIC 20-02-2013); Charles University, Prague, Czech Republic (15-28541A); Erasmus MC, Rotterdam, The Netherlands (MEC-2013-026); University Hospital Ghent, Belgium (B670201629681/B670201214983) and St. Anne's University, Brno, Czech Republic (METC 1G2015)-.

**Table 1:** Characteristics of patients, WBC and lymphocytes subsets (TBNK) reported by the referring clinician (x 10e3/μl).

**Category**	**Case no**.	**Gender**	**Disease**	**Mutation**	**Protein**	**WBC**	**T-cells (abs)**	**B-cells (abs)**	**NK-cells (abs)**	**Age**
SCID	Case_1	F	SCID, CD3E deficiency	CD3E exon 6 c.173delT	p.Leu58HisfsX9	13.9	0.32	2.6	1.4	0.3
SCID	Case_2	M	SCID, ADA deficiency	ADA exon 4 homozygous c.302G > A	p.Arg101Gln	2.5	0.03	0	0.01	1.3
SCID	Case_3	M	SCID, JAK3 deficiency with mat.engr.	JAK3 heterozygous c.561delT, c.2066C > T	p.Val188SerfsX14, p.Pro689Leu	11.8	6.31	3.02	0.02	1.8
SCID	Case_4	F	SCID, JAK3 deficiency	JAK3 exon 12 homozygoot c.1765G > A (NM_000215)	p.Gly589Ser	6.5	0.03	0.8	0.08	0
SCID	Case_5	F	SCID, JAK3 deficiency with mat.engr.	JAK3 exon 5 c.578G > A, exon 19 c.2712C > A	p.Cys193Tyr	15.5	1.68	0.37	0.1	0.2
SCID	Case_6	M	SCID, Cernunnos/XLF deficiency	NHEJ1 exon 5 homozygoot c.532C > T	p.Arg178X	3.4	0.22	0.04	0	0.9
SCID	Case_7	F	SCID, Artemis deficiency	DCLRE1C c.1A > C c.401C > G (compound heterozygote)	M1V, T134R (Met1Val, Thr134Arg)	3.6	0.00132	0	0.0018	0.4
SCID	Case_8	M	SCID, RAG2 deficiency	RAG2 homozygous c.1280_1281insTGGATAT	p.Asn428GlyfsX12	33.1	0.04	0.01	1.61	0.2
SCID	Case_9	M	SCID, RAG2 deficiency	RAG2 c.107G > A	p.Trp36*	2.8	0.04236	0.0444	0.0678	0.2
SCID	Case_10	M	Omenn syndrome, RAG1 deficiency	RAG1 c.983G > A/c.1186C > T (compound heterozygote)	pCys328Tyr/pArg396Cys	27.9	6.767	0.0303	1.818	0
SCID	Case_11	M	Omenn syndrome, RAG1 deficiency	RAG1 exon 2 c.519del	p.Glu174Serfs*27	8.05	2.15	0	0.429	0.3
SCID	Case_12	M	SCID, IL2RG deficiency with mat.engr.	IL2RG c.270-1G > A	n.d.	3.6	0.04	0.57	0	0.7
SCID	Case_13	M	SCID, IL2RG deficiency	IL2RG c.613G > A	p. Trp174*	9.5	0.001045	0.22325	0.022895	0.7
SCID	Case_14	M	SCID, IL2RG deficiency with mat.engr.	IL2RG c.269+3A > T	n.d.	5.4	0.6804	0.783	0.00891	0.5
SCID	Case_15	M	SCID, IL2RG deficiency	IL2RG exon 5 hemizygoot c.595-1G > T	n.d.	8.6	0	0.6	0.02	0.3
other PID	Case_16	M	CID, PNP deficiency	PNP c.700C > T	p.Arg234X	6.1	0.5	0.07	0.01	1.6
other PID	Case_17	M	ZAP70 deficiency	ZAP70 exon 10 homozygoot c.1193C > T	p.Ile398Ser	11.3	2.18	1.03	0.17	0.6
other PID	Case_18	M	Wiskott-Aldrich syndrome	WAS c. 1271_1295del	p.Gly424Glufs*13	8.4	1.47	0.95	0.25	0.3
other PID	Case_19	M	Wiskott-Aldrich syndrome	WAS c.344A > G	p.His115Arg	4	1.271	0.4305	0.3075	1.1
other PID	Case_20	M	Complete DiGeorge syndorme	del22q11.2		6.7	0.48776	0.003819	0.1206	1.6
other PID	Case_21	F	Complete DiGeorge syndorme	del22q11.2		5.1	0.00126684	0.655	0.504	0.2
other PID	Case_22	F	DiGeorge syndrome	del22q11.2		9.9	0.914354	1.342852	1.008826	0.6
other PID	Case_23	M	DiGeorge syndrome	del22q11.2		6.7	0.97	1.29	0.7	0.3
other PID	Case_24	M	Takenouchi-Kosaki syndrome	CDC42 c.191A > G	p.Tyr64Cys	2.4	0.436	0.094	0.094	1.5
other PID	Case_25	M	IPEX syndrome	FOXP3 c.721T > C	S241P (p.Ser241Pro)	15.135	2.42353	1.013115	0.349624	0.2
other PID	Case_26	M	Autoimmune lymphoproliferative sy	FAS exon 7 heterozygous (frameshift)	n.d.	29.8	20.818	2.146	0.226	0.3

### SCID-RTE Tube Composition and Staining Protocol

The SCID-RTE tube aims to assess relevant lymphoid subpopulations important in PID diagnostics in a single 8 color test. It includes markers for T-cells (CD3, CD4, CD8, TCRγδ), including their naïve (CD62Lpos, CD45ROneg) and RTE (CD31pos) stages, as well as their activated forms (HLA-DR). Detailed composition and volumes of antibodies used are listed in Table 2.

**Table 2:** Composition of the EuroFlow SCID-RTE tube^*^.

**Marker**	**Fluorochrome**	**Clone**	**Source**	**Catalog number**	**μl/test**
CD3	APC	SK7	BD Biosciences	345767	2.5
CD4	BV510	OKT4	Biolegend	317443	1.5
CD8	APC-Alexa750	B9.11	Beckman Coulter	A94683	1.5
CD31	PE	MEM-05	Exbio	1P-273-T100	5
CD45RO	FITC	UCHL1	Exbio	1F-498-T100	10
CD62L	BV421	DREG-56	Biolegend	304827	2
HLA-DR	PerCP-Cy5.5	L243	Biolegend	307629	1.5
TCRγδ	PE-Cy7	11F2	BD Biosciences	649806	2.5

* *Both the SCID-RTE tube and the PIDOT tube have originally be designed for application in 8-color format. However, because of their strong complementarity, it can be efficient and cost-effective to use a 12-color “combined PIDOT & SCID-RTE variant” by supplementing the PIDOT tube with the CD45RO, CD31, HLA-DR, and CD62L markers*.

The samples were processed in four EuroFlow laboratories (Charles University, Prague, Czech Republic; Erasmus MC, Rotterdam, The Netherlands; Ghent University, Ghent, Belgium; University of Salamanca, Salamanca, Spain) following standardized EuroFlow approaches (29, 30) (detailed protocols are publicly available at www.EuroFlow.org). In short, peripheral blood (*n* = 66) or cord blood (*n* = 4) (up to 2 ml) was mixed with ammonium chloride lysing solution (48 ml) and incubated for 15 min at room temperature in order to lyse erythrocytes. Obtained WBC were washed twice with phosphate buffered saline (PBS) containing 0.5% bovine serum albumin (BSA) and 0.09% sodium azide (NaN_3_) and subsequently stained with the antibodies listed in Table 2 in a final volume of 100 μl for 30 min at room temperature in the dark. Whenever possible, up to one million cells were processed, or all cells available in lymphopenic PID patients. In each case, at least 2 × 10^5^ cells were stained. Next, the cells were incubated with 2 ml BD FACS™ Lysing Solution (BD Biosciences) for 10 min at room temperature in the dark, washed and resuspended in 250 μl washing solution.

### Data Acquisition and Analysis

Data acquisition was performed on BD FACSCanto II, BD LSR II or BD FACSLyric instruments (BD Biosciences) equipped with 405, 488, and 633/640 nm lasers and PMT detectors, following the EuroFlow instrument set-up Standard Operating Protocol (29, 31). Data were analyzed using Infinicyt (Cytognos, Salamanca, Spain) and FlowJo (FlowJo LLC, Ashland, Oregon) software. Normal values for all T-cell subsets were determined as numbers above the 5th percentile of the healthy controls. In case of HLA-DR positive activated T-cells, we determined normal values as below the 95th percentile of the healthy controls (see Table 3). Therefore, specificity was by definition 95%. For sensitivity calculations we divided the number of patients with an abnormal value by the total number of patients measured in each parameter/subset separately (see Table 3). For statistical analysis, GraphPad Prism software Mann-Whitney test was used.

**Table 3:** Lymphocytes subsets evaluated by SCID-RTE tube.

**Patient code**	**Disease**	**Category**	**CD3+ of Lym**	**CD3+ abs**	**TCRgd+ of T**	**TCRgd+ abs**	**CD4+ T of T**	**CD4+T abs**	**CD8+ T of T**	**CD8+T abs**	**CD4+RTE of CD4**	**CD4+RTE abs**	**Naive CD4+ of CD4**	**Naive CD4+ abs**	**Naive CD8+ of CD8**	**Naive CD8+ abs**	**HLA-DR+ of CD4**	**HLA-DR+ CD4+ abs**	**HLA-DR+ of CD8**	**HLA-DR+ CD8+ abs**
Case_1	CD3E	SCID	**7**	**246**	0	0	**5**	**13**	93	**228**	**0**	**0**	**0**	**0**	**2**	**5**	**93**	12	**83**	**188**
Case_2	ADA	SCID	55	**18**	**13**	2	**1**	**0**	83	**15**	**0**	**0**	**0**	**0**	**0**	**0**	**n/a**	**n/a**	**93**	14
Case_3	JAK3	SCID	69	5,610	1	67	**4**	**210**	94	5,256	**0**	**0**	**0**	**1**	**2**	**117**	**64**	**134**	**95**	**4,967**
Case_4	JAK3	SCID	**2**	**15**	3	0	**1**	**0**	85	**12**	**0**	**0**	**0**	**0**	**0**	**0**	**n/a**	**n/a**	**99**	12
Case_5	JAK3	SCID	80	2,247	0	4	98	2,202	**1**	**23**	**0**	**0**	**0**	**1**	**0**	**0**	**86**	**1,896**	**86**	19
Case_6	XLF	SCID	**38**	**294**	**89**	263	**0**	**1**	**2**	**7**	**0**	**0**	**0**	**0**	**0**	**0**	**n/a**	**n/a**	**64**	5
Case_7	Artemis	SCID	**18**	**84**	**55**	46	**38**	**31**	**1**	**1**	**6**	**2**	**6**	**2**	**31**	**0**	**88**	28	**44**	0
Case_8	RAG2	SCID	**2**	**40**	1	1	71	**29**	**1**	**0**	**0**	**0**	**0**	**0**	**17**	**0**	**91**	26	**n/a**	**n/a**
Case_9	RAG2	SCID	**32**	**78**	5	4	86	**67**	**5**	**4**	**0**	**0**	**0**	**0**	**3**	**0**	**66**	44	**77**	3
Case_10	RAG1	SCID	68	4,621	3	158	**43**	1,964	44	2,024	**0**	**0**	**0**	**0**	**0**	**1**	**85**	**1,661**	**86**	**1,739**
Case_11	RAG1	SCID	69	**1,916**	6	109	88	1,690	**3**	**64**	**0**	**0**	**0**	**2**	**1**	**1**	**68**	**1,154**	**83**	53
Case_12	IL2RG	SCID	**12**	**92**	0	0	71	**65**	28	**25**	**0**	**0**	**0**	**0**	**4**	**1**	**94**	61	**81**	20
Case_13	IL2RG	SCID	**0**	**1**	5	0	**31**	**0**	**1**	**0**	**0**	**0**	**0**	**0**	**0**	**0**	**n/a**	**n/a**	**n/a**	**n/a**
Case_14	IL2RG	SCID	**45**	**677**	**25**	171	**48**	**325**	23	**156**	**0**	**0**	**1**	**3**	**7**	**11**	**87**	**282**	**94**	**147**
Case_15	IL2RG	SCID	**0**	**1**	**17**	0	58	**0**	**0**	**0**	**0**	**0**	**0**	**0**	**0**	**0**	**n/a**	**n/a**	**n/a**	**n/a**
Case_16	PNP	other PID	63	**281**	3	7	**48**	**134**	37	**105**	**0**	**0**	**0**	**0**	**0**	**0**	**63**	**84**	**99**	**103**
Case_17	ZAP70	other PID	**51**	**1,661**	3	47	89	1,482	**2**	**29**	**24**	**357**	**34**	**505**	**19**	**5**	**17**	**246**	**30**	9
Case_18	WAS	other PID	**50**	**1,092**	4	41	82	**891**	**13**	**138**	**53**	**470**	82	**730**	**56**	**78**	3	23	3	4
Case_19	WAS	other PID	57	**978**	**28**	271	**31**	**303**	38	376	**18**	**55**	**40**	**122**	**6**	**23**	**39**	**119**	**85**	**319**
Case_20	del22q11.2	other PID	**50**	**328**	**37**	122	**15**	**47**	**3**	**9**	**0**	**0**	**0**	**0**	**1**	**0**	**43**	21	**65**	6
Case_21	del22q11.2	other PID	**0**	**1**	**10**	0	**0**	**0**	70	**1**	**0**	**0**	**0**	**0**	**14**	**0**	**n/a**	**n/a**	**58**	1
Case_22	del22q11.2	other PID	**27**	**1,592**	**9**	138	65	**1,030**	22	344	**56**	**579**	**71**	**729**	87	300	**5**	56	4	14
Case_23	del22q11.2	other PID	**33**	**756**	**8**	63	65	**493**	22	**169**	**50**	**246**	72	**356**	87	**147**	**4**	20	4	7
Case_24	CDC42	other PID	63	**465**	**39**	180	**34**	**159**	23	**107**	**40**	**63**	**66**	**105**	**58**	**63**	**6**	10	**21**	22
Case_25	FOXP3	other PID	61	**1,664**	1	22	70	**1,157**	22	363	**40**	**465**	**66**	**765**	81	295	**4**	48	5	19
Case_26	FAS	other PID	86	12,833	3	**444**	**26**	3,324	15	1,912	58	1,938	81	2,692	92	1,761	**11**	**356**	5	**88**
**Controls**																				
5th percentile			**52**	**2,010**	0.7	20	**54**	**1,201**	**13**	**335**	**58**	**840**	**72**	**1,030**	**60**	**287**	0	9	0	2
95th percentile			86	6,626	**7**	**340**	82	4,094	35	2,204	86	2,393	96	3,149	96	1,614	**3**	**66**	**11**	**80**
Sensitivity other PID			55%	91%	55%	20%	55%	82%	27%	64%	91%	91%	73%	91%	64%	73%	90%	40%	55%	27%
Sensitivity SCID			67%	80%	33%	60%	60%	80%	53%	87%	**100%**	**100%**	**100%**	**100%**	**100%**	**100%**	**100%**	50%	**100%**	33%

*Absolute counts (abs) as 10e3/μl. Values outside the normal range are in bold. Range obtained in controls (5th and 95th percentile) is given below the table. Sensitivity to disclose abnormal values in other PID and SCID group is given for each measurement in the bottom two rows. Most informative parameters are highlighted in gray*.

## Results

### Composition of the SCID-RTE Tube

**Figure 1:**
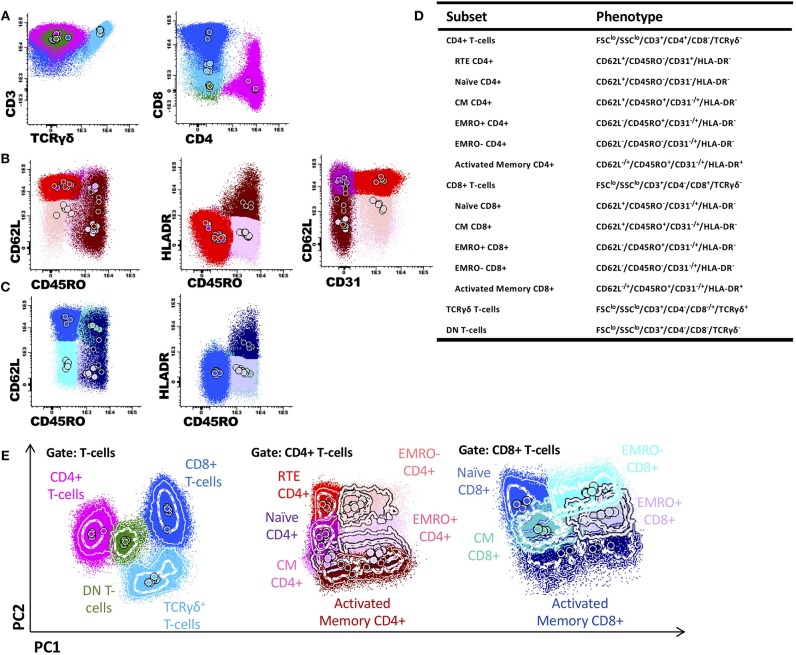
Gating T-cell subsets and generation of a reference principal component analysis representation in an n-dimensional space for SCID-RTE tube. **(A)** After gating T-cells as CD3+ and FSC^lo^ and SSC^lo^, the markers TCRγδ+, in combination with CD4 and CD8 were used to define TCRγδ+ T-cells (light blue), CD4+CD8-TCRγδ- T-cells (pink); CD4-CD8+TCRγδ- T-cells (dark blue) and CD4-CD8- TCRγδ- double negative T-cells (green). **(B)** The CD4+ T-cell subsets were further subdivided into recent thymic emigrants (RTE; CD62L+CD45RO-HLDR-CD31+; red), naïve (CD62L+CD45RO-HLDR-CD31-; purple), central memory (CM; CD62L+CD45RO+HLDR-; orchid), effector memory CD45RO+ (EMRO+; CD62L-CD45RO+HLDR-; mauve), effector memory CD45RO- (EMRO-; CD62L-CD45RO-HLDR-; pink) and activated memory (CD45RO+HLDR+; burgundy) CD4+ T cells. **(C)** The CD8+ T-cell maturation subsets were further subdivided into naïve (CD62L+CD45RO-HLDR-; blue), central memory (CM; CD62L+CD45RO+HLDR-; blue-green), effector memory CD45RO+ (EMRO+; CD62L-CD45RO+HLDR-; periwinkle blue), effector memory CD45RO- (EMRO-; CD62L-CD45RO-HLDR-; cyan) and activated memory (CD45RO+HLDR+; navy blue) CD8+ T cells. **(D)** Definition and hierarchy of the defined subsets. **(E)** Principal component analysis representation (APS view) based on the most discriminating parameters for T-cell populations, and CD4+ T-cells and CD8+ T-cell subsets.

The SCID-RTE tube was designed with the purpose of identifying the relevant lymphoid subpopulations important in PID diagnostics of severe PID in newborns, using a single 8-color test. It identifies naïve CD4+ T cells and among them, the RTEs. The definition of naïve T-cells includes a selection of non-activated (HLA-DR negative) cells, together with absence of CD45RO (a memory T-cell marker) and the presence of the naïve T-cell marker L-selectin (CD62L). The definition of RTE uses the naïve T-cell gate and is further complemented by CD31, Platelet endothelial cell adhesion molecule (PECAM-1) (Table 2, Figure 1). After gating T-cells as CD3+ and lymphocytes on FSC and SSC, four markers (CD3, TCRγδ, CD4, and CD8) were used to define TCRγδ+ and TCRγδ- CD4+, CD8+ and double negative (DN) T-cells (Figure 1A, Supplemental Figure 1). The CD4+ T-cells were further subdivided into RTE, naïve, central memory (CM), effector memory CD45RO+ (EMRO+) and CD45RO- (EMRO-) and activated memory T-cells (Figures 1B,D); for CD8+ T-cells the same subsets were defined except for the RTEs (Figures 1C,D). The total set and hierarchy of T-cell subsets that was identified is listed in Figure 1D. To offer intuitive and fast interpretation of the complete lymphoid compartment we developed a new analysis and visualization strategy for the SCID-RTE tube using principle component analysis (PCA)-based multidimensional views (APS graphs). First, reference plots were generated using a set of 10 samples of healthy donors in Infinicyt software. The lymphocyte populations were manually analyzed and subsequently, the most discriminating projection into a single APS graph was determined (Figure 1E). A software tool for automated identification of the cell populations present in the SCID/RTE tube was built, containing normal blood samples stained with the same antibody combination.

### Identification of RTEs by the SCID-RTE Tube in PID Patients

**Figure 2:**
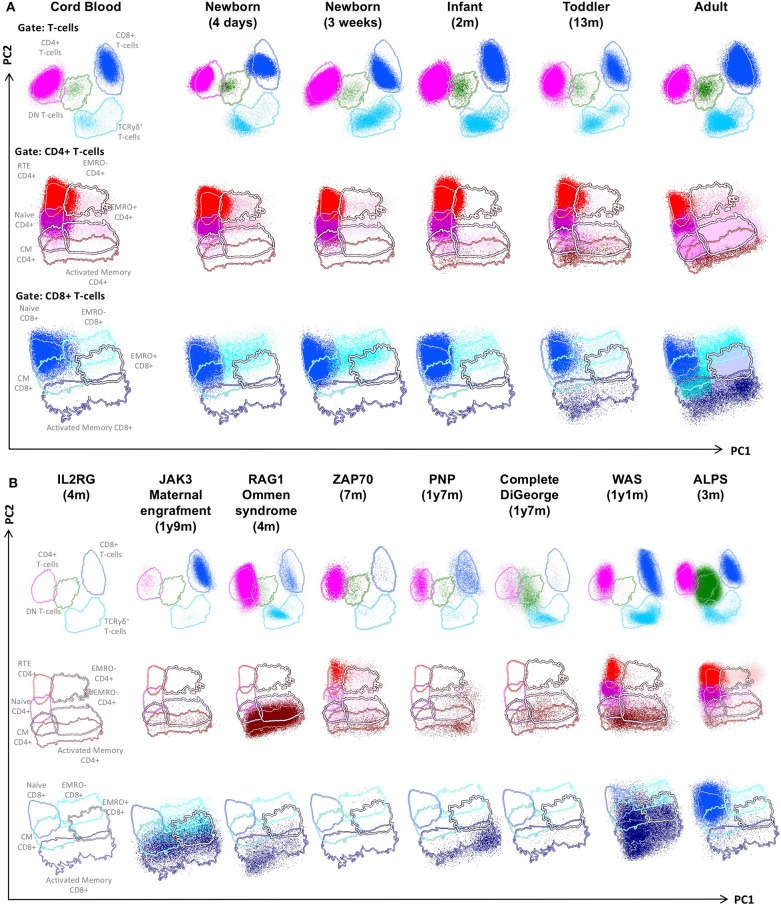
PCA representation of SCID-RTE tube results, showing the distinct blood T-cell subsets in the supervised PCA analysis of blood samples from healthy donors of different age **(A)** and SCID and CID patients **(B)**. From the top down, APS plots of gated total, CD4+ and CD8+ T-cells are shown. Lines depict a 2 standard deviation boundary of all controls combined. **(A)** PCA (APS views) of all T-cell subsets of cord blood and peripheral blood from donors of different age. **(B)** PCA (APS views) of all T-cell subsets of the following patients: a IL2RG-deficient patient, a JAK3-deficient patient with maternal engraftment, a RAG1-deficient Omenn syndrome, a ZAP70-deficient patient, a PNP-deficient patient, a complete DiGeorge syndrome, a Wiskott-Aldrich syndrome (WAS) and an autoimmune lymphoproliferative syndrome (ALPS) patient.

**Figure 3:**
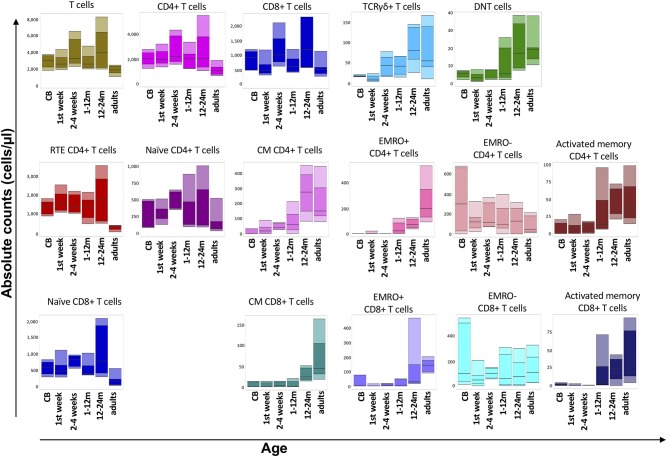
Flow cytometric analysis of T-cell populations using the EuroFlow SCID/RTE tube in 56 healthy controls of five different age ranges. All values of this reference data set are displayed as bar graphs representing the median, and p10, p25, p75, and p90 percentiles. For data visualization package gplot2 for the statistical language R was used.

The SCID-RTE tube allows analysis of the naïve and memory subsets of T-cells (Figure 2A) that are abnormally distributed in patients with PID (Figure 2B). Typically, their numbers are different in childhood compared to the adult age (Figure 3), however within 2 years of age a general threshold for CD4+ RTE (<800 cell/μl), naïve CD4+ (<1,000 cell/μl) and naïve CD8+ lymphocytes (<290 cell/μl) is justified. Notably, the number of RTEs are abundant in childhood, whereas the numbers of activated memory CD4+ and CD8+ T-cells are low (Figures 2A, 3).

SCID patients that present without T-cells (Figure 2B, *IL2RG*) are straightforwardly identified by the SCID-RTE tube, but they never pose a diagnostic dilemma even in a simple T-B-NK flow cytometric assay. However, in SCID and Omenn syndrome patients with paradoxically normal or even increased total absolute numbers of T-cells, the SCID-RTE tube allows detection of the activation status of T-cells (HLA-DR positive). These activated T-cells can be of maternal origin in SCID with maternal engraftment (Figure 2B, *JAK3 with maternal engraftment*) or can be oligoclonal, expanded T-cells in Omenn syndrome patients (Figure 2B, *RAG1 Omenn syndrome*). RTE cells are virtually absent in these patients with T-cell production defects (SCID and Omenn patients). Patients with PNP deficiency and complete DiGeorge syndrome also lack RTEs, but patients with ZAP70 deficiency, Wiskott-Aldrich Syndrome and ALPS have detectable RTEs (Figure 2B, detailed dot plots shown in Supplemental Figures 2, 3).

### Absence of RTEs in SCID Patients

In our current study, we analyzed 15 SCID patients and 11 other PID patients diagnosed before 2 years of age. SCID patients had disease-causing mutations in *RAG1 or RAG2* (*n* = 4, two of them presented with Omenn syndrome), IL2RG (*n* = 4, one of them with confirmed maternal engraftment), *NHEJ1* (*n* = 1), *CD3E* (*n* = 1), *ADA* (*n* = 1), *JAK3* (*n* = 3, two of them with maternal engraftment) and *DCLRE1C* (*n* = 1) (see Table 2 and Supplemental Figure 2).

In the SCID patients, the absolute levels of CD3+ T-cells were strongly reduced (*n* = 7) or undetectable (*n* = 5), but for three patients (20% of our cohort) the absolute CD3+ T-cell counts were in the normal range (see Table 3). These patients were proven to have maternal engrafted T-cells (*n* = 2) or oligoclonally expanded cells characteristic for Omenn syndrome (*n* = 1).

**Figure 4:**
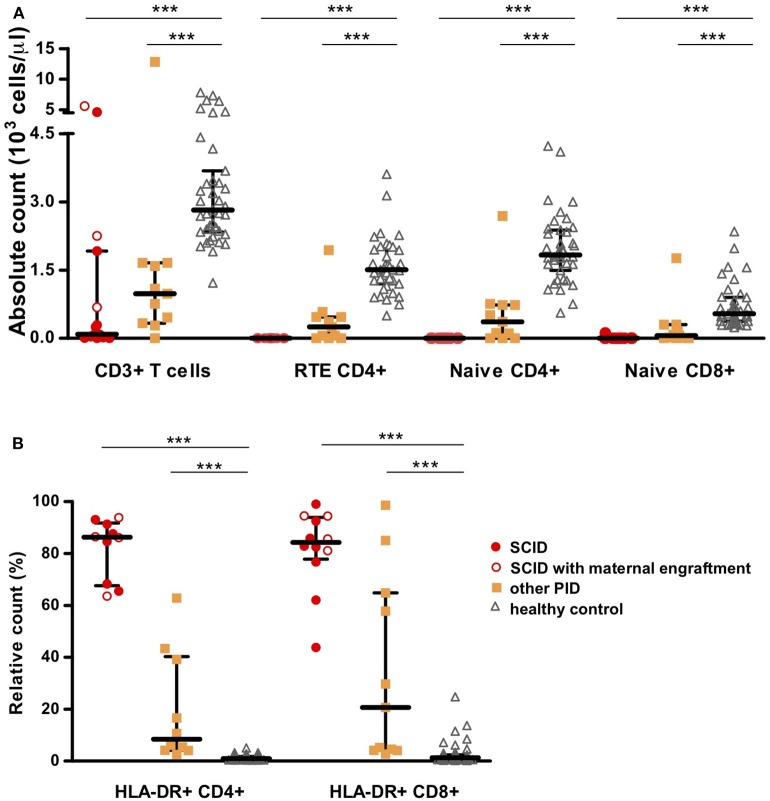
T-cell subset counts determined using the EuroFlow SCID-RTE tube. **(A)** Absolute values of CD3+ T-cells, RTE CD4+ cells, naïve CD4+ or CD8+ T-cells. **(B)** Relative values of activated T-cells based on the expression of HLA-DR molecule on CD4+ T-cells (HLA-DR+CD4+) or CD8+ T-cells (HLA-DR+CD4+). SCID patients (*n* = 15) are represented as red circles where open circles show patients with maternal engraftment, other PID patients (*n* = 11) as orange squares and healthy controls (*n* = 44) as gray triangles. Detailed gating strategy is shown in Supplemental Figure 1. ***P ≤ 0.0005.

Application of the SCID-RTE tube showed that all SCID patients completely lacked RTE cells and other forms of naïve CD4+ and CD8+ T cells (Figure 4A), even the patients with normal T-cell counts (due to maternal T-cells or oligoclonal expansion). The T-cells that could be detected had signs of massive activation (64–94% HLA-DR+ in CD4+ and 44–99 % in CD8+ T cells) (Figure 4B). Overall, the SCID-RTE tube detected severely decreased or absent numbers of RTE and naïve CD4+ and CD8+ T-cell subsets in all SCID patients. In the SCID patients with detectable levels of T-cells, the phenotype was characterized by activation (HLA-DR+) and had a memory phenotype (CD45RO+).

### RTEs in Other Severe PID Diagnosed Before 2 Years of Age

Patients diagnosed with other severe forms of PID (Other PID, *n* = 11) had diverse molecular defects [*ZAP70* (*n* = 1), *WAS* (*n* = 2), *PNP* (*n* = 1), *FOXP3* (*n* = 1) del22q11.2 (DiGeorge *n* = 2; complete DiGeorge *n* = 2), *CDC42* (*n* = 1) and *FAS* (*n* = 1)] (see Table 2 and Supplemental Figure 3). Except for the patient with autoimmune lymphoproliferative syndrome (ALPS) due to FAS mutation, all had decreased absolute counts of CD3+ T-cells compared to controls. However, only one complete DiGeorge patient had <300 T-cells/μl in the conventional TBNK test, which is considered a diagnostic threshold in SCID patients.

When assessed by the SCID-RTE tube the patients showed a heterogeneous pattern of T-cell subset abnormalities ranging from strongly reduced/absent to normal numbers. However, all of the patients showed at least one abnormality. With the exception of the ALPS patient who presented with normal proportion of naïve CD4+ T-cells (and elevated naïve CD8+ T-cell counts) (32), all had reduced naïve CD4+ T-cells and RTEs below 5th percentile of healthy (Figures 2B, 4A, Table 3). The ALPS patient was characterized by massively increased T-cells especially of double negative T-cells (56% of CD3+TCRγδ- cells) and activated CD4+ T-cells (11%). As previously described (33), we found a high frequency of TCRγδ+ T cells (9–39 % of CD3+ cells), in patients with Wiskott-Aldrich syndrome (WAS) as well as in DiGeorge (*n* = 2) and complete DiGeorge patients. We also identified a high frequency of TCRγδ+ T-cells in CDC42 deficiency. Four had normal total CD8+ T-cell counts, but reduced naïve CD8+ T-cells and showed signs of activation: WAS, immune dysregulation, polyendocrinopathy, enteropathy, X-linked syndrome (IPEX), DiGeorge (Table 3).

On top of reduction or absence of naïve and RTE subsets of CD4+ T-cells (Table 3), three of the four DiGeorge patients showed decreased CD8+ T cells and their naïve subsets. Activation of T cells (as measured by HLA-DR+) ranged from mild to high. Both patients with WAS had profoundly reduced naïve CD8+ T-cells, reduced naïve CD4+ T-cells and RTE, activation was found in both CD4+ and CD8+ T cells in one of the WAS patients. The complete dataset of findings for all patients is provided in Table 3.

### Added Value of SCID-RTE on Top of the PIDOT Tube

**Figure 5:**
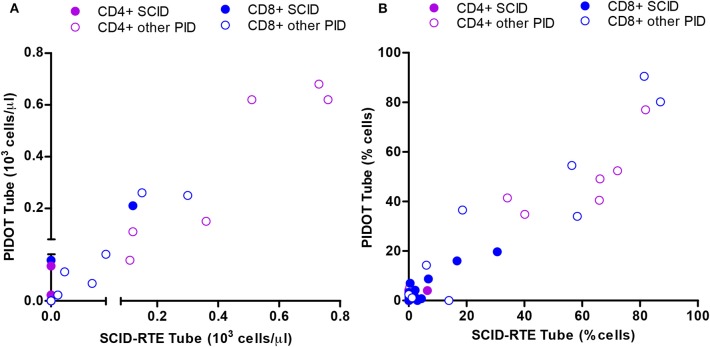
Correlation between the SCID-RTE and the PIDOT tube in determining the levels of naïve CD4+ and CD8+ T-cell subsets in PID patients. **(A)** Absolute counts or **(B)** Relative counts for each PID patient (*n* = 24) of naïve CD4+ T-cells (blue) and naïve CD8+ T-cells (green) measured by the SCID-RTE tube (x axis) and the PIDOT tube (y axis). SCID (closed circles), other PID (open circles) patients. Detailed gating strategy is shown in Supplemental Figure 1.

The proposed SCID-RTE tube is both a confirmation and extension of the PIDOT tube. As the reduction of naïve T-cells was one of the most important hallmarks of (S)CID and PID in our cohort, as well as in the large group of PID patients published previously (18), we investigated whether the definition of naïve T-cells in the PIDOT tube (CD45RA+CD27+) corresponds to the SCID-RTE tube definition (CD45ROnegCD62L+HLA-DRneg). Indeed, we found that both approaches yield correlating values in the PID patients (Figure 5). Thus, the PIDOT tube is capable of detecting reduction of naïve T-cells and directing the testing toward confirmation with the SCID-RTE tube, which also allows the specific detection of RTEs and activation status, which is particularly important in patients with normal or close to normal T-cell counts.

Both the PIDOT tube and the SCID-RTE tube have originally been designed for application in an 8-color format. However, because of their strong complementarity, it can be efficient and cost-effective to use the 12-color combined PIDOT & SCID-RTE variant in cases suspicious of (S)CID, by supplementing the PIDOT tube with the CD45RO, CD31, HLA-DR, and CD62L markers.

### Specificity and Sensitivity

Next, we determined the sensitivity of the SCID-RTE tube to find abnormal values (defined as below the 5th percentile, thus allowing for specificity 95%) in our cohort of 15 (S)CID and 11 other PID patients.

The sensitivity of values of naïve CD4+, CD8+, and CD4+ RTE yielded 100% sensitivity to detect (S)CID (see Table 3). This was despite the fact that several (S)CID patients presented with close to normal total T-cells and their basic subsets (CD4+ and CD8+ T-cells). Whenever T-cells were detectable, their activation status (percentage of HLA-DR) was an equally sensitive marker for (S)CID, as the naïve T-cells and RTEs.

However, naïve T-cells and RTEs were also abnormal in the group of other PID, implying that these patients could also be recognized and referred for genetic testing. Thus, it can be concluded that in case of reduced naïve T-cells and RTEs fast genetic testing is urgently needed.

## Discussion

There have been multiple initiatives for establishing comprehensive and detailed reference values of human lymphocyte subsets in children by several groups (34–36). These studies were done by using two to four color flow cytometry. There was no attempt to standardization and no clear indication about the utility of individual subsets' abnormalities for PID diagnostics. Recently, Takashima et al. reported on a detailed set of seven 8–10 color flow cytometry panels used to investigate 75 PID patients, where they also found lack of naïve T-cells in SCID (also with maternal engraftment), Ataxia Telangiectasia and CMCD (37). However, this 7-tube approach would be demanding to use at large scale, and it requires relatively high sample volume for the seven tube-aliquots. With the EuroFlow PID consortium, we developed a standardized approach for flow cytometry testing in PID (38), which includes a tube for screening and orientation (PIDOT) (18), two 8-color tubes for analysis of pre- and post-germinal center B-cells, and an additional isotype tube allowing full characterization of B-cells, including analysis of IgH isotype and subclass distribution within the memory B-cell (MBC) and plasma cell (PCs) compartments (39). The EuroFlow approach offers a systematic approach to diagnostics with a modular design (37).

Here we focused on the feasibility and performance characteristics of the EuroFlow SCID-RTE tube for diagnostic use in (S)CID and severe PID in a cohort of 26 patients, genetically diagnosed before the age of 2 years. The challenge in revealing SCID patients comes from the fact that a large portion of them presents with detectable T-cells that are either autologous oligoclonal T-cells as seen in Omenn syndrome (40, 41), or arise from maternal engraftment [40% of SCID according to Mueller et al. (19)]. The EuroFlow SCID-RTE tube overcomes the limitations of the basic T, B, NK test that cannot evaluate the nature of the T-cell subsets. In particular, the CD4+ naïve, CD8+ naïve, and CD4+ RTE subsets are shown to be decreased in all SCID and a great majority of other PID in our cohort. The RTE subset was reported as a useful proxy for thymic output measurements (25, 42–45), correlating to TREC levels (43, 45–47), that are used in newborn screening programs for SCID.

Since (S)CID patients harbor deleterious mutations that prevent normal T-cell development, any T-cells in their bloodstream must be expanded T-cells of either autologous oligoclonal origin or maternal origin. In order to improve the RTE definition for patients with putative peripheral expansion of T-cells, we gated not only on CD31+CD45RO-CD4+ T-cells, but we additionally excluded TCRγδ+ and HLA-DR positive cells and restricted the gate to include CD62L positive cells only. Thus, only naïve, non-activated CD4+ T-cells (non-TCR γδ+) are counted as RTE. This improved the accuracy of the RTE measurements, particularly in other PID patients with massive presence of HLA-DR, where some activated (HLA-DR+) cells would be otherwise considered RTE. HLA-DR was reported as a marker of residual T-cells in Omenn syndrome patients by Saint Basil et al. (27) already in 1991. The biological significance and mode of HLA-DR acquisition by T-cells is thought to be explained by acquisition of the molecule from antigen presenting cell (APC) after T-cell-APC contact (48).

A difficult PID category to be diagnosed with the SCID-RTE tube would be DiGeorge syndrome patients who present with near-normal counts of T-lymphocytes and their subsets. DiGeorge patients have variable clinical presentations, TREC levels and T-cell counts that are generally lower than normal, but vary considerably from patient to patient (14). However, their clinical course rarely requires HSCT. Rare cases of complete DiGeorge patients resembled SCID patients in their immunophenotype and thus pose no diagnostic challenge. While in other PID patients in our cohort, T-cell production (thymopoiesis) is not the mechanism responsible for the immunodeficiency, but their mutation leads to more complex changes broadly termed as dysregulation (WAS, ALPS, IPEX, ZAP70, CID), the SCID-RTE tube was also able to find abnormalities, mainly in the naïve/RTE compartments and in the T cell subset activation status. A CDC42 mutation in a Takenouchi-Kosaki syndrome (49, 50) patient was accidentally found in an infant with failure to thrive, lymphopenia and lymphedema by whole exome sequencing for PID suspicion. Abnormalities in lymphoid cells were clearly revealed by the PIDOT and SCID-RTE tube.

The SCID-RTE tube can also be used in patients with Combined Immunodeficiency (CID), where severe clinical presentation together with laboratory findings indicative of CID can be used for HSCT indication. ESID criteria for CID diagnosis require that apart from severe infection or immune dysregulation or affected family members, two of the four following T-cell criteria must be met: (a) reduced CD3 or CD4 or CD8 T-cells, (b) reduced naïve CD4 and/or CD8 T-cells, (c) elevated TCRγδ+T-cells, (d) reduced proliferation to mitogen or TCR stimulation (51). All three immunophenotypic criteria can be readily obtained from the SCID-RTE tube, furthermore the threshold counts of CD4+ RTE (<800 cell/μl), naïve CD4+ (<1,000 cell/μl) and naïve CD8+ lymphocytes (<290 cell/μl) are established in mutli-center and standardized diagnostic test. We would propose that the SCID-RTE tube can be used whenever there is a high clinical suspicion for SCID or CID. The SCID-RTE tube should be measured together with the PIDOT tube to obtain insight in the lymphocytes' compartment and to screen and diagnose (S)CID in a fast, standardized and efficient manner. It can also be used in a sibling of a SCID patient, immediately after birth or in children with low or absent TRECs as identified via newborn screening. SCID-RTE and PIDOT can yield the required information confirming severe T-cell abnormality or disproving it in a pre-symptomatic phase, but a separate study is needed to validate this approach in a newborn screening program. SCID-RTE can be used in patient where some abnormalities in the T-cell compartment were found by PIDOT tube. Finally, SCID-RTE and PIDOT can serve as a complementary immunophenotyping test for patients with positive TREC findings, where immunophenotyping information can serve to confirm the diagnosis of PID and direct subsequent genetic testing. Moreover, it can offer hints for the decision making process on appropriate conditioning regimen before HSCT. Importantly, the two 8-color SCRID-RTE and PIDOT tubes can also be combined into a single 12-color tube for more efficient testing.

In conclusion, we have shown that the EuroFlow SCID-RTE tube is a well-performing, fast and standardized diagnostic test for (S)CID that can be deployed in any laboratory with 8-color flow cytometer.

## Data Availability Statement

The datasets generated for this study are available on request to the corresponding author.

## Ethics Statement

The study was approved by the local ethics committees of the participating centers [University of Salamanca, Salamanca, Spain (USAL-CSIC 20-02-2013); Charles University, Prague, Czech Republic (15-28541A); Erasmus MC, Rotterdam, The Netherlands (MEC-2013-026); University Hospital Ghent, Belgium (B670201523515) and St. Anne's University, Brno, Czech Republic (METC 1G2015)]. The ethics committee waived the requirement of written informed consent for participation.

## Author's Note

All authors wish to stress that they are scientifically independent and have full freedom to act without any obligation to industry other than scientific advice to companies in the context of licensed patents. The selection of antibodies by the EuroFlow consortium is always explicitly based on quality, relevance, and continuous availability. Consequently all proposed antibody panels consist of mixtures of antibodies from many different companies.

## Author Contributions

MBu, TK, AO, and JD contributed to the conception and design of the study. TK, MBa, MBl, MP-A, BB, VK, CB, JP, EB, IP-K, JHMPP, BW-K, MP, JT, FH, HA, RF, TF, MS, AŠ, and SA-M performed the data acquisition and data analysis. MBa, TK, and MBu wrote the manuscript. All authors contributed to manuscript revision, read and approved the submitted version.

### Conflict of Interest

JD, AO, MBu, MP-A, TK, and EB each report being one of the inventors on the EuroFlow-owned patent PCT/NL 2015/ 050762 (Diagnosis of primary immunodeficiencies). The Infinicyt software is based on intellectual property (IP) of some EuroFlow laboratories (University of Salamanca in Spain and Federal University of Rio de Janeiro in Brazil) and the scientific input of other EuroFlow members. All above mentioned intellectual property and related patents are licensed to Cytognos (Salamanca, ES), which pays royalties to the EuroFlow Consortium. These royalties are exclusively used for continuation of the EuroFlow collaboration and sustainability of the EuroFlow consortium. JD and AO report an Educational Services Agreement from BD Biosciences (San José, CA) and a Scientific Advisor Agreement with Cytognos; all related fees and honoraria are for the involved university departments at Leiden University Medical Center and University of Salamanca, respectively. The remaining authors declare that the research was conducted in the absence of any commercial or financial relationships that could be construed as a potential conflict of interest.

## Supplementary Material

Supplemental Figure 1 Detailed gating strategy.

Supplemental Figure 2 Detailed flow cytometry dot plots for SCID patients.

Supplemental Figure 3 Detailed flow cytometry dot plots for other PID patients.
