# Supplementary figures and images for: EuroFlow Standardized Approach to Diagnostic Immunopheneotyping of Severe PID in Newborns and Young Children
Source: Front Immunol. 2020 Mar 19;11:371. doi: 10.3389/fimmu.2020.00371 (PMC7096355; doi:10.3389/fimmu.2020.00371)

Gating strategy EuroFlow SCID-RTE tube

Ungated

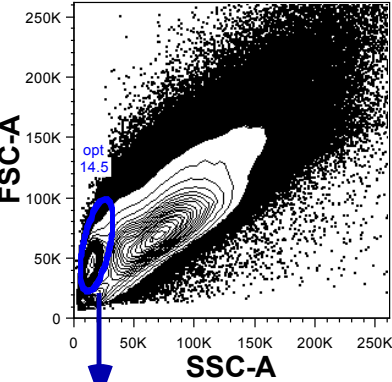

singlets

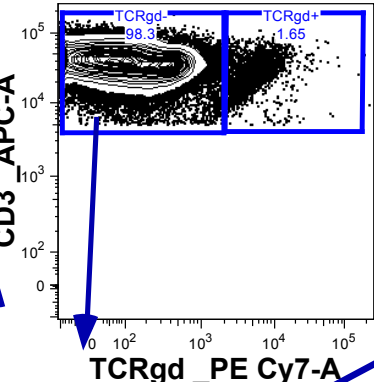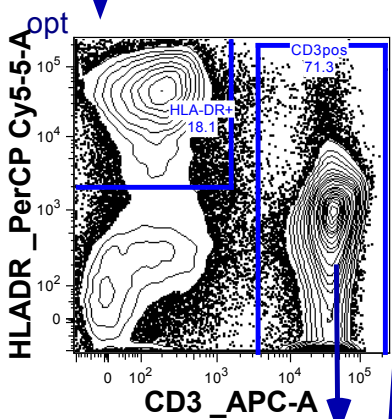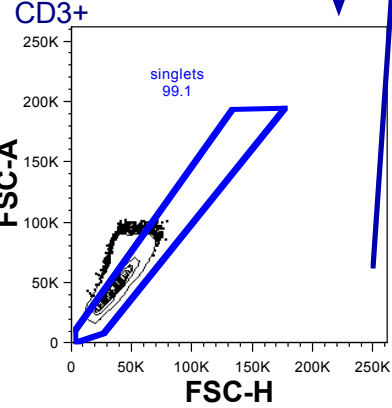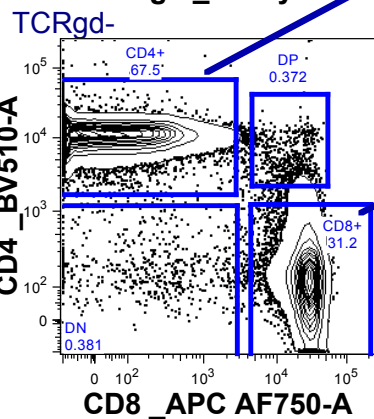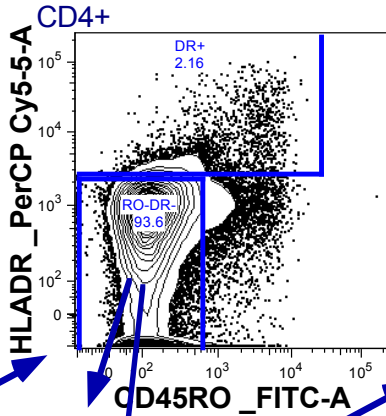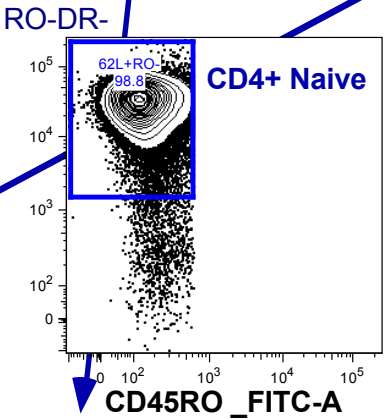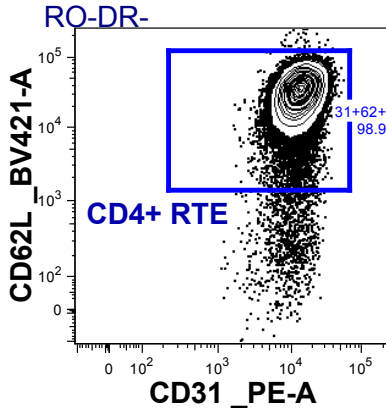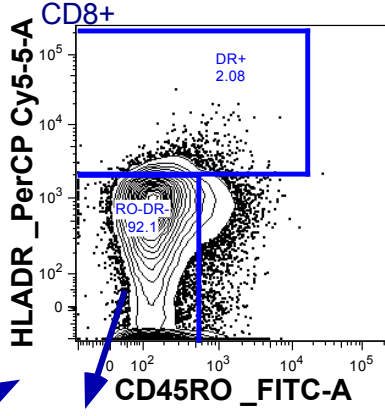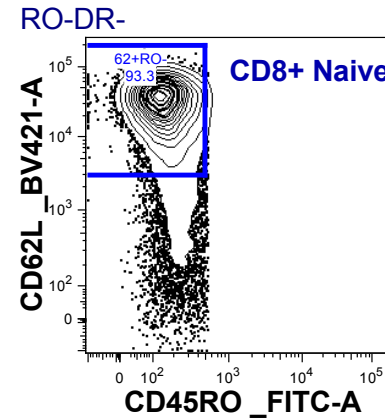

Supplement: Supplemental Figure 1 — Detailed gating strategy. [file Data_Sheet_1.PDF]

# RAG1 (Omenn syndrome)

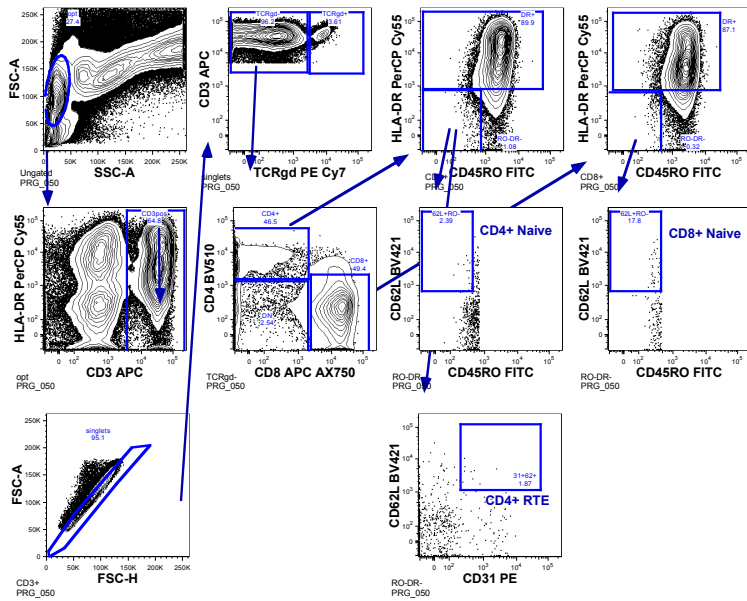

# RAG2

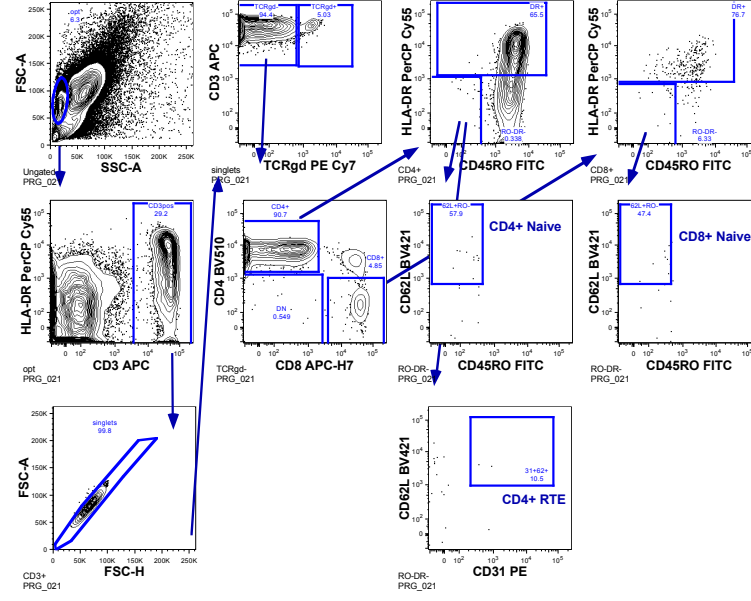

# IL2RG

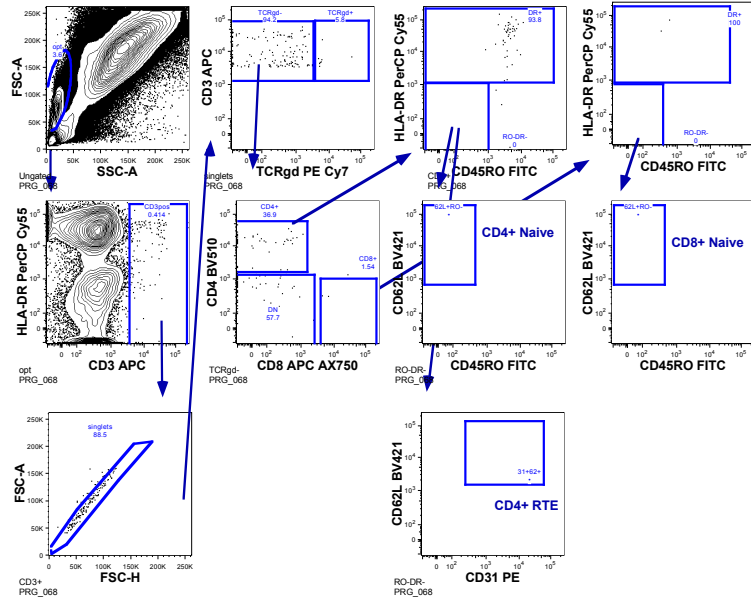

# IL2RG (maternal engraftment)

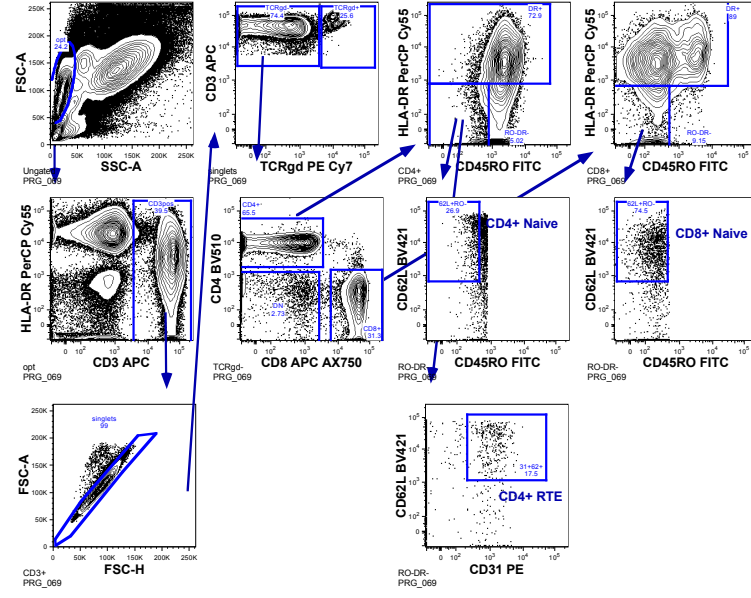



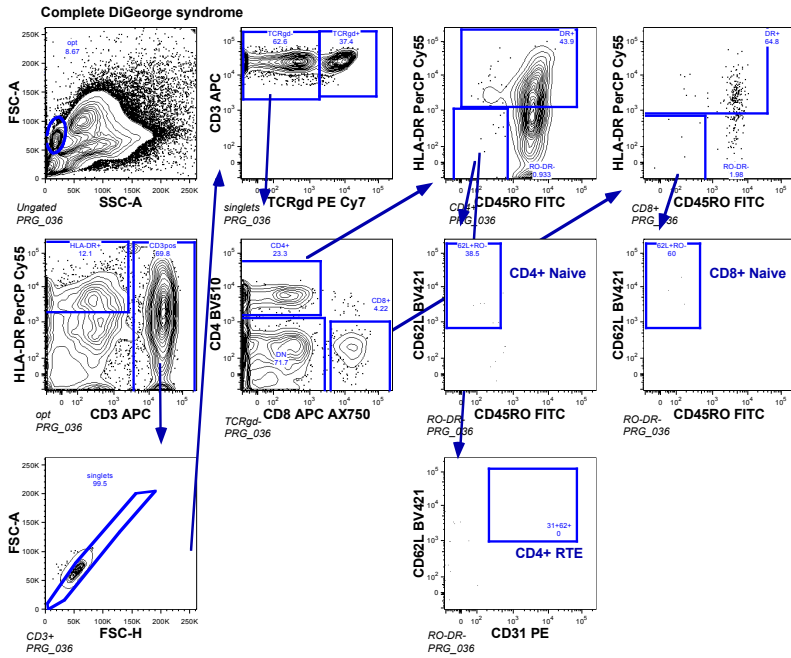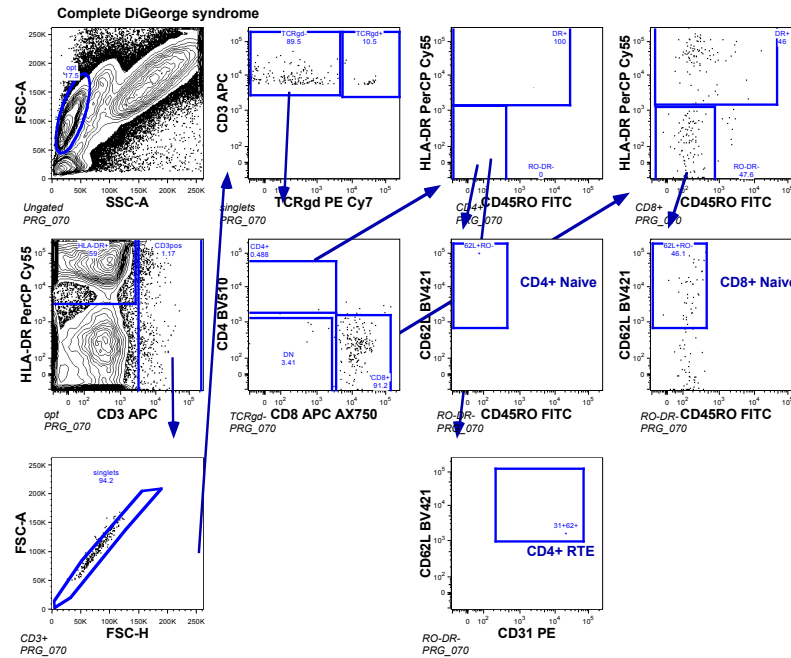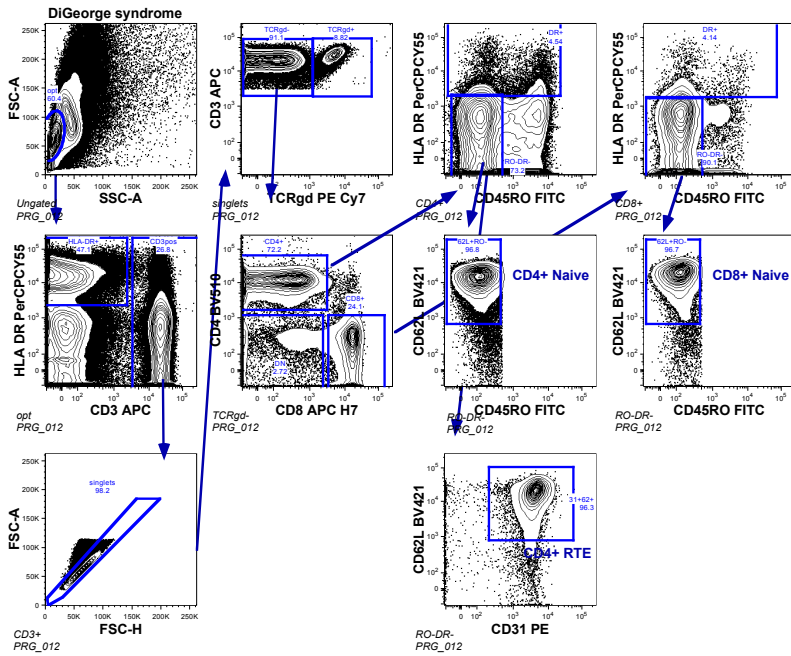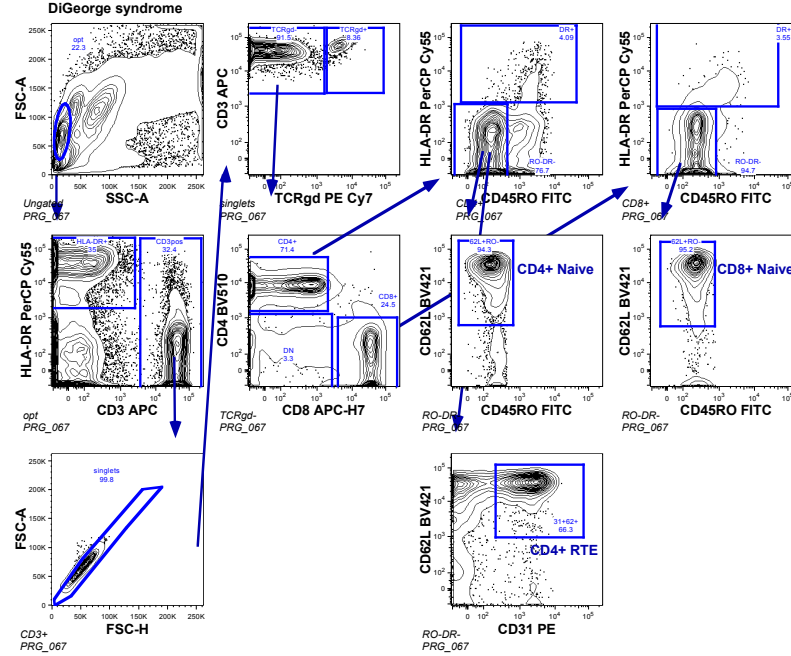

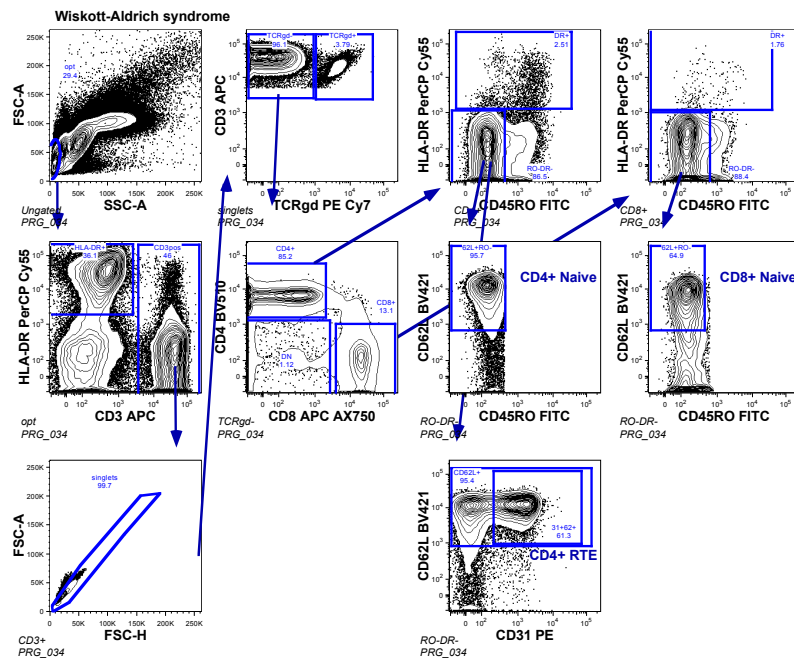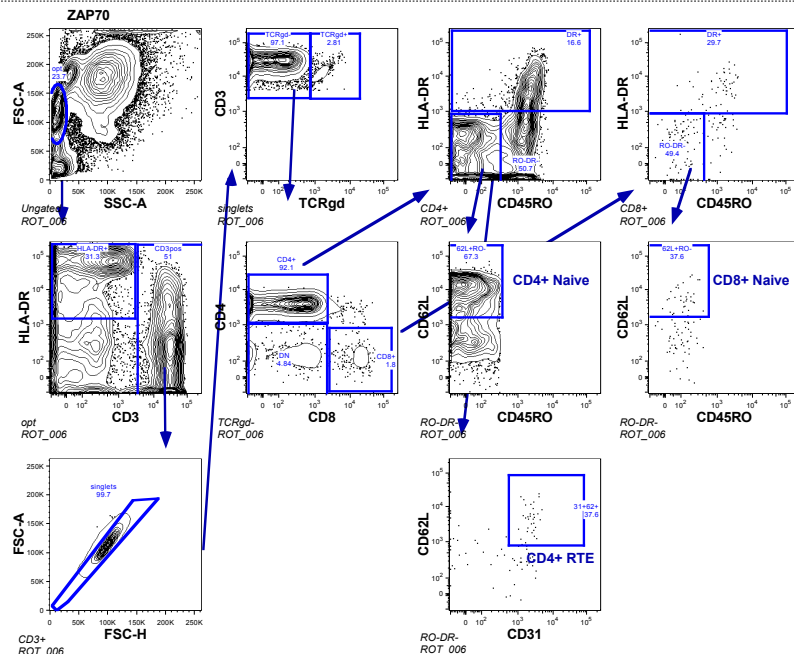

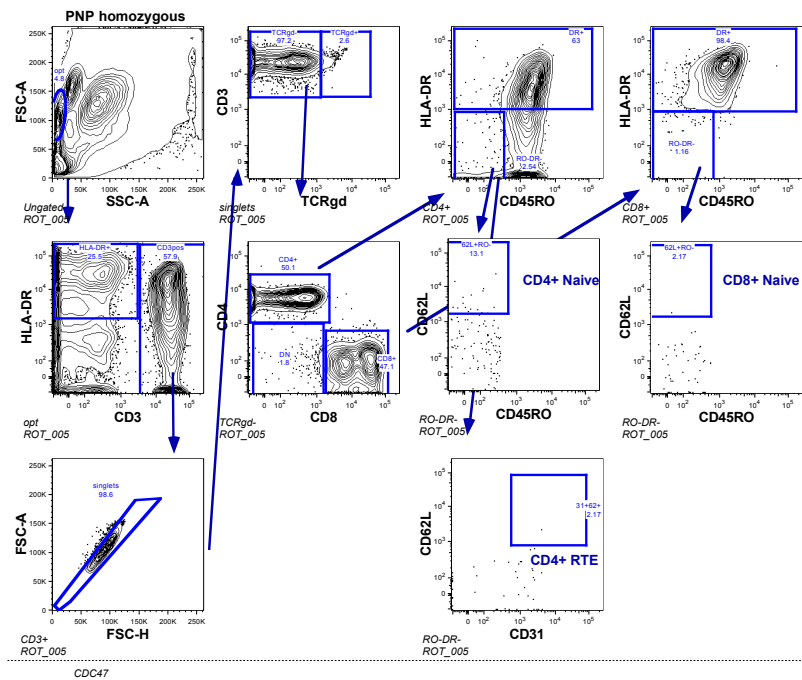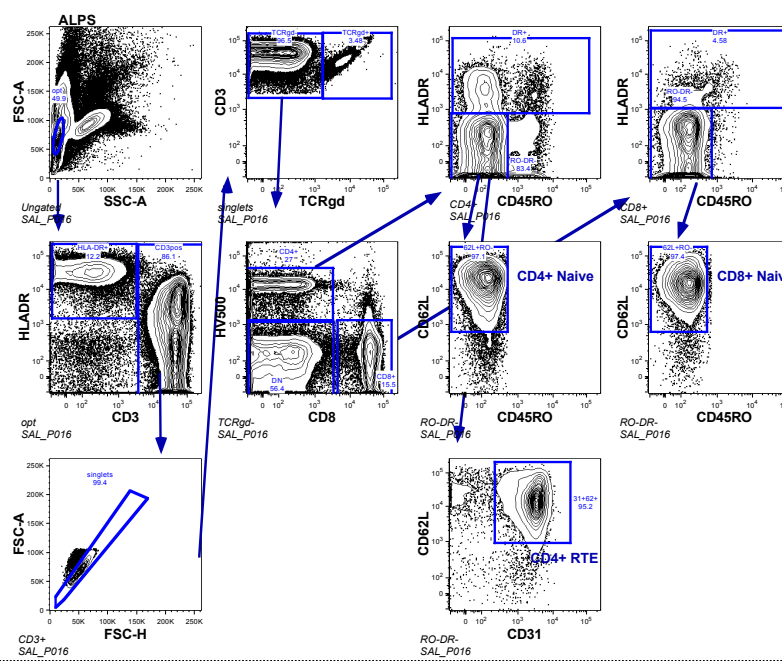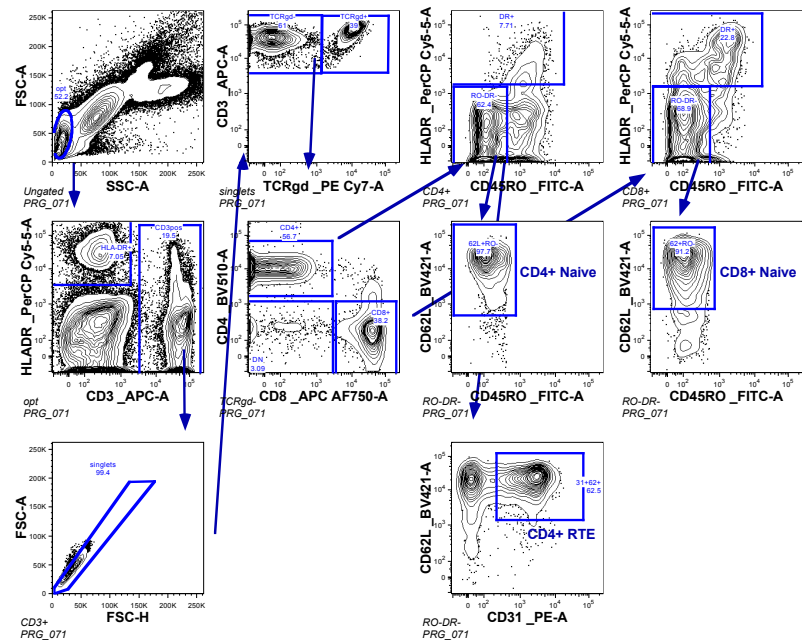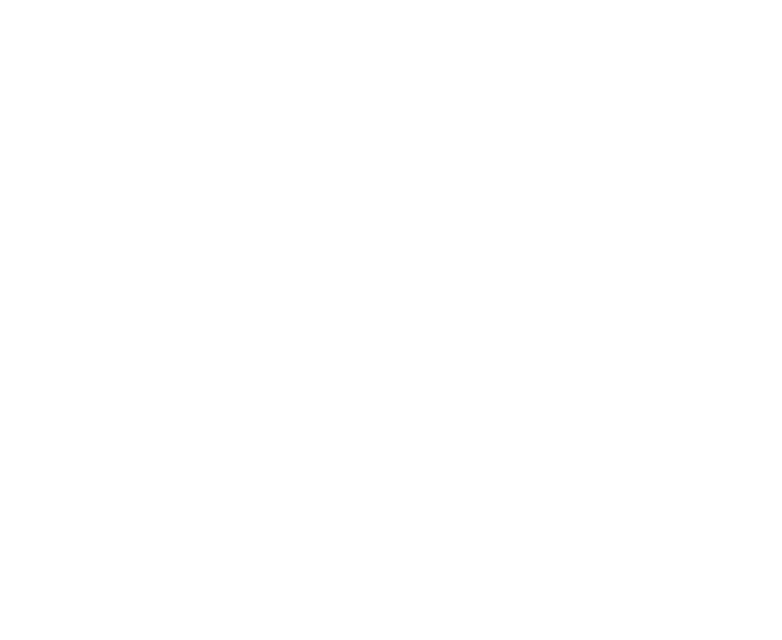

Supplement: Supplemental Figure 2 — Detailed flow cytometry dot plots for SCID patients. [file Data_Sheet_2.PDF]

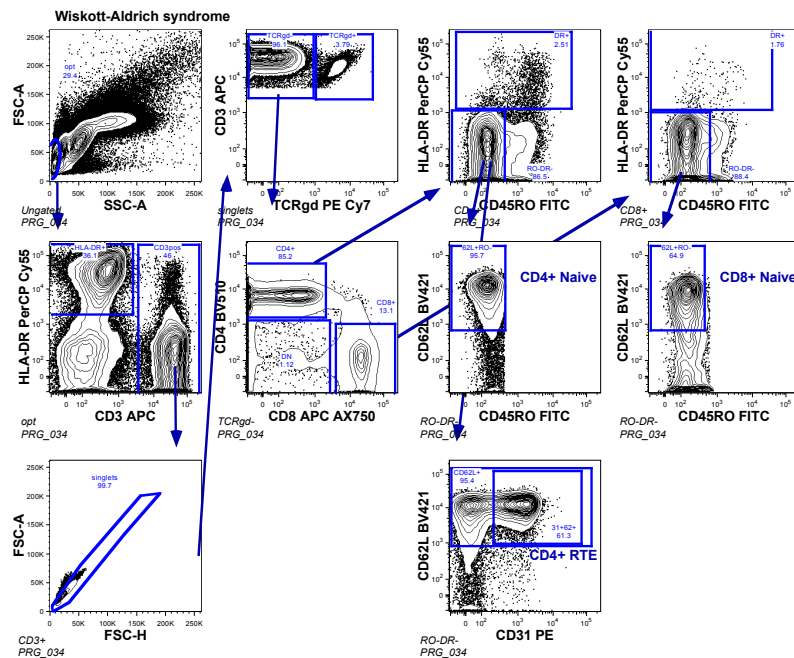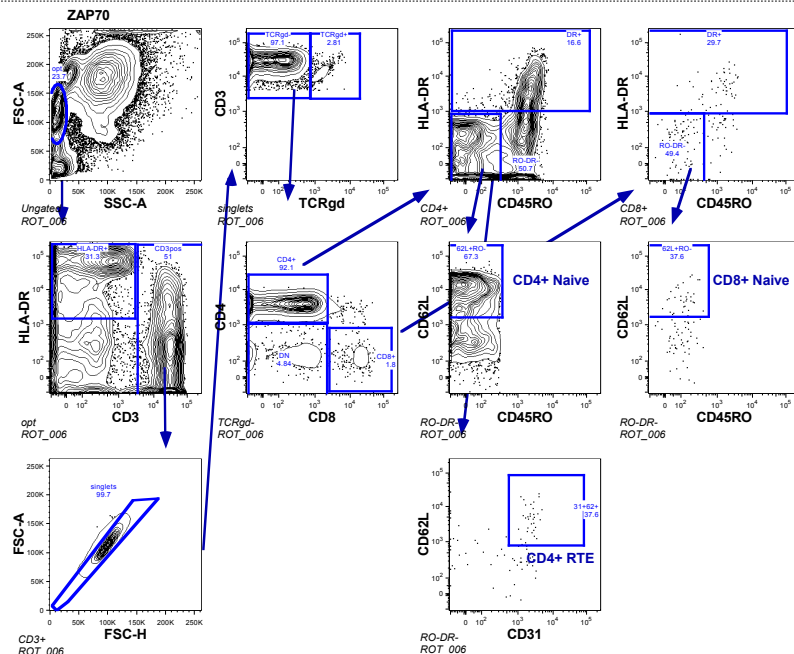

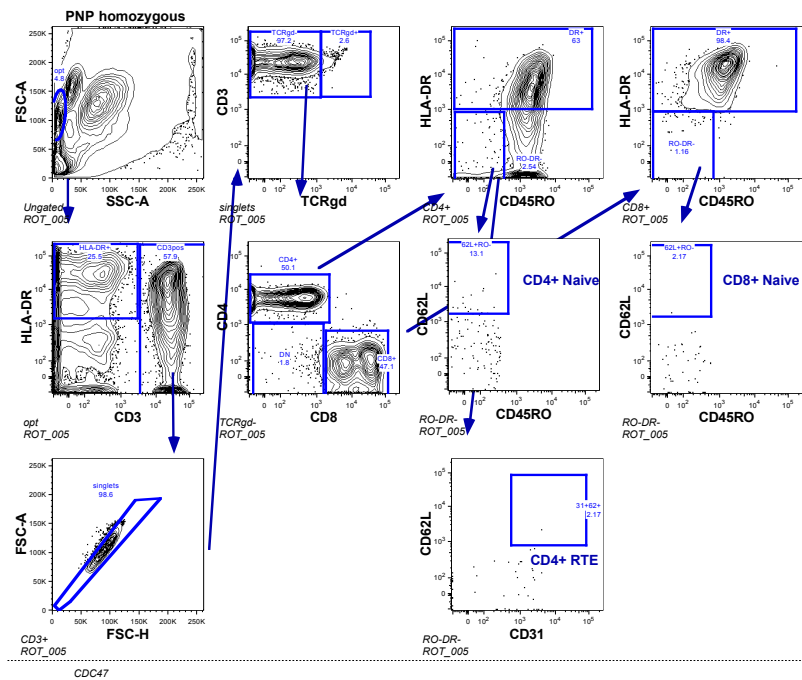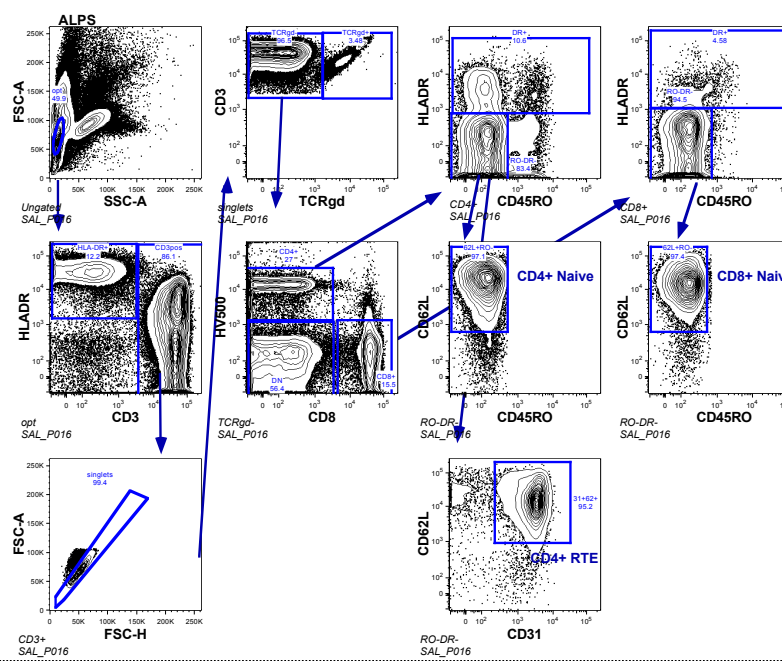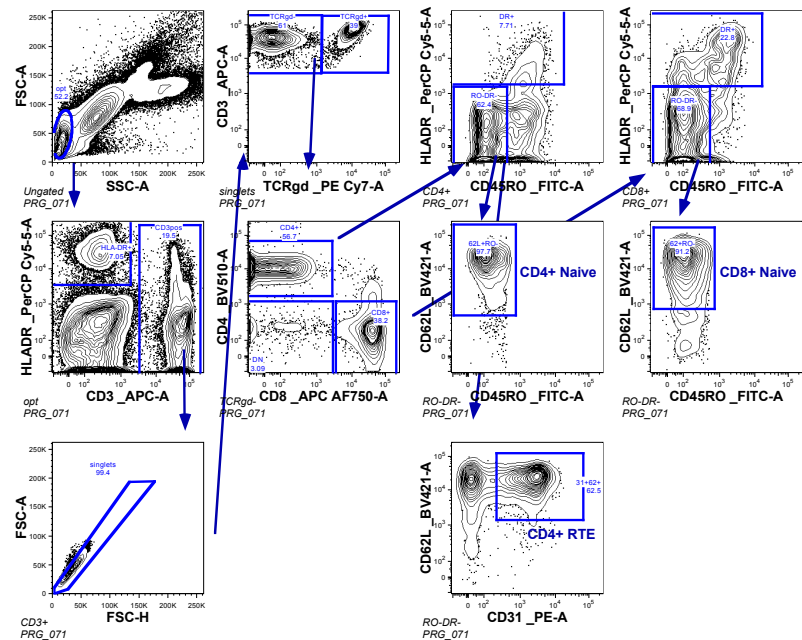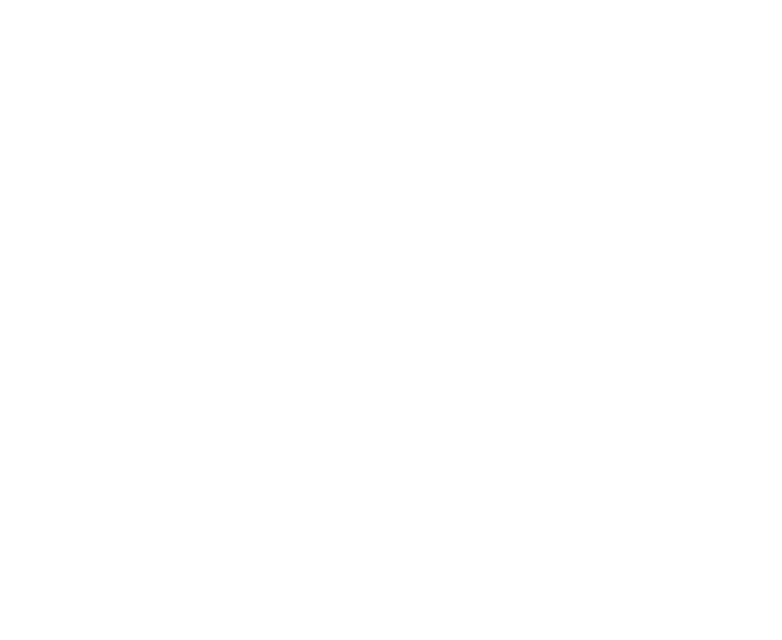

Supplement: Supplemental Figure 3 — Detailed flow cytometry dot plots for other PID patients. [file Data_Sheet_3.PDF]
